# Do Individuals Skimp on Health Care After Spouse’s Dementia Diagnosis?

**DOI:** 10.1093/geroni/igab046.061

**Published:** 2021-12-17

**Authors:** Yi Chen

**Affiliations:** University of Southern California, Irvine, California, United States

## Abstract

Dementia is a costly disease that places great burden on individuals and families. The substantial time and financial resources taken away by living with persons with dementia (PWDs) may make their spouses forgo essential health care, thus deteriorating long-term health and increasing downstream healthcare costs. However, such negative externality is understudied. This paper studied the impacts of spouse's incident dementia diagnosis on an individual's use of needed care, defined as annual flu shot and regular doctor visits for those with preexisting conditions. Using HRS linked to Medicare claims, I employed a fixed effects approach to compare the use of flu shot and doctor visit during 1 year before and after the index, for individuals whose spouse had dementia (N=691) and otherwise similar controls (N=5,073). After adjusting for time-varying health, caregiving roles, and other socio-demographic factors, spouse’s dementia onset was associated with greater likelihood of getting flu shot and seeing doctors. Among those transitioning into caregiving, spouses of PWDs had a marginally higher risk of skimping on doctor visits, compared to controls (p=0.053). In this broadly representative sample, there lacks evidence for rationed health care ensuing spouse’s dementia incidence, at least within a 1-year time frame. However, for new spousal caregivers, the impact of dementia is more profound and complex than deprivation of time. This group may face a trade-off between caring for spouses with dementia and caring for themselves, for whom policy support merits further study and consideration.

